# Benefits of E-Cigarettes Among Heavy Smokers Undergoing a Lung Cancer Screening Program: Randomized Controlled Trial Protocol

**DOI:** 10.2196/resprot.4805

**Published:** 2016-02-03

**Authors:** Claudio Lucchiari, Marianna Masiero, Giulia Veronesi, Patrick Maisonneuve, Stefania Spina, Costantino Jemos, Emanuela Omodeo Salè, Gabriella Pravettoni

**Affiliations:** ^1^Università Degli Studi di MilanoDepartment of PhilosophyMilanoItaly; ^2^Università Degli Studi di MilanoHealth SciencesUniversità Degli Studi di MilanoMilanoItaly; ^3^European Institute of OncologyThoracic DevisionMIlanItaly; ^4^European Institute of OncologyDivision of Epidemiology and BiostatisticsMIlanItaly; ^5^European Institute of OncologyApplied Research Unit for Cognitive and Psychological ScienceMIlanItaly; ^6^European Institute of OncologyDivision of PharmacyMilanItaly; ^7^Università Degli Studi di MilanoDepartment of Oncology and Hemato-oncologyMilanItaly

**Keywords:** tobacco cessation, electronic cigarettes, lung cancer screening, smoking related diseases.

## Abstract

**Background:**

Smoking is a global public health problem. For this reason, experts have called smoking dependence a global epidemic. Over the past 5 years, sales of electronic cigarettes, or e-cigarettes, have been growing strongly in many countries. Yet there is only partial evidence that e-cigarettes are beneficial for smoking cessation. In particular, although it has been proven that nicotine replacement devices may help individuals stop smoking and tolerate withdrawal symptoms, e-cigarettes’ power to increase the quitting success rate is still limited, ranging from 5% to 20% dependent on smokers’ baseline conditions as shown by a recent Cochrane review. Consequently, it is urgent to know if e-cigarettes may have a higher success rate than other nicotine replacement methods and under what conditions. Furthermore, the effects of the therapeutic setting and the relationship between individual characteristics and the success rate have not been tested. This protocol is particularly innovative, because it aims to test the effectiveness of electronic devices in a screening program (the COSMOS II lung cancer prevention program at the European Institute of Oncology), where tobacco reduction is needed to lower individuals’ lung cancer risks.

**Objective:**

This protocol was designed with the primary aim of investigating the role of tobacco-free cigarettes in helping smokers improve lung health and either quit smoking or reduce their tobacco consumption. In particular, we aim to investigate the impact of a 3-month e-cigarettes program to reduce smoking-related respiratory symptoms (eg, dry cough, shortness of breath, mouth irritation, and phlegm) through reduced consumption of tobacco cigarettes. Furthermore, we evaluate the behavioral and psychological (eg, well-being, mood, and quality of life) effects of the treatment.

**Methods:**

This is a prospective, randomized, placebo-controlled, double-blind, three-parallel group study. The study is organized as a nested randomized controlled study with 3 branches: a nicotine e-cigarettes group, a nicotine-free e-cigarettes group, and a control group. The study is nested in a screening program for early lung cancer detection in heavy smokers.

**Results:**

The study is open and is still recruiting.

**Conclusions:**

Stopping or reducing tobacco consumption should be a main goal of any health organization. However, traditional antismoking programs are expensive and not always effective. Therefore, favoring a partial or complete shift to e-cigarettes in heavy smokers (eg, persons at high risk for a number of diseases) could be considered a moral imperative. However, before following this path, sound and reliable data on large samples and in a variety of contexts are required.

**Trial Registration:**

Clinicaltrials.gov NCT02422914; https://clinicaltrials.gov/ct2/show/NCT02422914 (Archived by WebCite at http://www.webcitation.org/6etwz1bPL)

## Introduction

The World Health Organization estimates that cigarette smoking will claim the lives of 500 million people who are alive today and as many as 1 billion people during the 21st century. Although clinical therapies for smoking cessation have proven effective, the long-term abstinence rate remains low.

Electronic cigarettes, which are also known as tobacco-free cigarettes or e-cigarettes, are battery-operated devices that vaporize a liquid solution of propylene glycol and/or vegetable glycerin in which nicotine and/or flavors are dissolved. A recent review of the field showed that e-cigarettes may be considered safe, with few adverse effects and limited toxicity [1].

The value of these tools is that they reduce the risk of smoking-related diseases. However, while the use of e-cigarettes in heavy smokers will reduce the risk of tobacco-related cancers, their role in antismoking programs has not yet been approved. The World Health Organization and the US Food and Drug Administration have promoted the launch of research on this field of study, but study results are not convergent.

In a prospective study, e-cigarettes were shown to substantially decrease the consumption of tobacco cigarettes without causing significant side effects [2]. In the study, reductions in the number of cigarettes smoked per day and breath carbon monoxide (CO) levels were observed at each visit in all study groups, with no consistent differences among them. Furthermore, rapid improvement in breathing symptoms was observed.

However, most participants continued smoking or started smoking again; after 1 year, fewer than 10% remained abstinent. This is probably due to the research targeting smokers who did not intend to quit. As suggested by Remo and colleagues [3], we argue that much better results might be achieved in smokers motivated to quit.

From a physiological point of view, e-cigarettes appear to eliminate the craving for tobacco in the same way as nicotine replacement therapy (NRT). In an overview of the Cochrane Library [4] that considered studies globally—including more than 50,000 smokers—NRT was described as being particularly efficacious for the short term (3 months) but less so in the long term (12 months). However, the use of NRT increases the success rate of quitting attempts independent of the setting if compared to attempts made by smokers on their own or supported only by counseling. Furthermore, NRT is particularly useful for smokers who are prepared to quit, but who have high nicotine dependence. Eventually, NRT will be particularly effective when smokers’ baseline conditions are predictors of successful quitting. Comparing the conclusion of the Cochrane review with the results of studies on e-cigarettes effects, it is clear that in some cases the sample used was quite different from the one used in most trials on NRT. We argue that it is necessary to study e-cigarettes efficacy while also considering population baseline characteristics as well as psycho-cognitive parameters. Indeed, we can expect smokers who are not prepared to quit or in psychosocial conditions associated with a low success rate (ie, mood disturbance, living in a context with high prevalence of tobacco cigarettes smokers, low-income status) to report a worse outcome in studies using e-cigarettes as tobacco cessation treatment [2,4].

Previous research has stressed that monitoring lifestyle parameters (in particular, physical activity and sleep quality) and acting on them could help maintain abstinence. In particular, physical exercise may aid smokers in the first 3 months, while longer effects are less clear [5]. It has been observed that regular physical activity among smokers reduces nicotine withdrawal symptoms and craving. Last, smoking during the night is an indicator of nicotine dependence and predicts failure in smoking cessation [6]. Sleep disturbances have several negative psychological effects, including reduced quality of life and psychological distress (ie, anxiety and depression). Low sleep quality due to abstinence is a predictor of a poor smoking treatment outcome [7].

Low-cost, noninvasive devices are now available to monitor lifestyle parameters. These electronic bracelets are reliable and easy to use. Counseling approaches based on similar tools have been shown to improve outcomes [8].

This protocol may also address a number of psychological parameters, in order to determine whether individual features might hamper behavioral changes and related positive effects on health. Indeed, heavy smokers have specific psycho-cognitive traits [9]. In particular, they generally show higher levels of impulsiveness than nonsmokers. At the same time, smokers tend to have a high level of activity in the behavioral activation system (BAS). It has been suggested that high BAS sensitivity is involved in addictive behaviors like smoking. Individuals with high BAS are more inclined to enact approaching behaviors and experience positive effects when they receive positive rewards.

This study offers an opportunity to test the effectiveness of e-cigarettes in a clinically controlled setting, in order to reduce tobacco consumption and improve health benefits. Furthermore, the protocol is nested in a screening program for early detection of lung cancer at the European Institute of Oncology (IEO) called COSMOS II (Continuous Observation of SMOking Subjects) that will allow subject recruitment and continuous monitoring. The COSMOS II project aims to improve early diagnosis of lung cancer, which is currently considered to be the most important life-saving tool. This is an Italian program, coordinated by IEO and created to identify an optimal personalized protocol for early diagnosis in people with a high risk of lung cancer (ie, heavy smokers or former smokers over the age of 55). The COSMOS II program will enroll 10,000 heavy smokers or former smokers throughout Italy. COSMOS II derives from the previous successful COSMOS I screening project [10,11].

The main hypothesis is based on previous research on the effect of e-cigarette use in substitution of tobacco cigarettes. We hypothesize that the reduction of cigarette tobacco consumption leads to a significant decrease of cough, breath shortness, and other respiratory symptoms at 6 months. This reduction will improve the quality of life and well-being. Furthermore, we expect this effect to be higher for smokers using nicotine e-cigarettes and support than for a placebo group and support-only group. This would be coherent with previous research on NRT and e-cigarettes. Indeed, we argue that smokers who are both motivated to start a quitting attempt and aware of smoking-related risks yet continue consuming several tobacco cigarettes a day need an integrated antismoking strategy that combines physiological, behavioral, and psychological interventions. The use of electronic cigarettes, providing both physiological and behavioral replacement of tobacco cigarette consumption, and low-intensity counseling, providing psychological support, might then be an optimal strategy in these cases.

Eventually, we expect particular psychological conditions to be associated with better outcomes. In particular, we hypothesize that participants with a low level of depression, an active lifestyle, and a low BAS will find the use of e-cigarettes more advantageous.

## Methods

### Objectives

The main objective was to evaluate the impact of a 3-month e-cigarettes program to reduce smoking-related respiratory symptoms (eg, dry cough, breath shortness, mouth irritation, and phlegm) as a consequence of reduced tobacco cigarette consumption. Secondary objectives were to (1) assess the success rate of smoking cessation attempts in the three groups; (2) monitor safety and toxicity during the study; (3) evaluate psychological and behavioral (ie, lifestyle) effects of e-cigarettes; (4) assess the impact of e-cigarette use on quality of life; and (5) identify cognitive/behavioral patterns as e-cigarettes success predictors in reducing tobacco cigarette smoking.

More specifically, the main aim of the project concerns the effectiveness of e-cigarettes in improving lung health in the heavy smokers involved in the COSMOS II program. If proven safe and effective, e-cigarettes should be included in lung cancer screening programs as a standard tool to reduce smoking-related risks for lung diseases. Naturally, this aim requires a scientific approach, since e-cigarettes should not increase nicotine dependence. Another fundamental aim of the project regards the effectiveness of e-cigarettes in reducing tobacco consumption. In particular, no studies to date have tested the feasibility and effectiveness of these tools in limiting risky behaviors (eg, tobacco smoking) among heavy smokers enrolled in a lung cancer screening program. Consequently, we want to determine whether providing e-cigarettes to participants in a controlled protocol reduces tobacco consumption, as well as related health and breathing problems. We also aim to analyze the psychological characteristics and needs of the subjects enrolled in the COSMOS II program, in order to evaluate how risk perception (eg, the premise of risky behavior adoption) is associated with a psycho-cognitive profile. We argue that an important and successful screening project, such as COSMOS, should incorporate a comprehensive approach to the individual.

### Design

**Figure 1:**
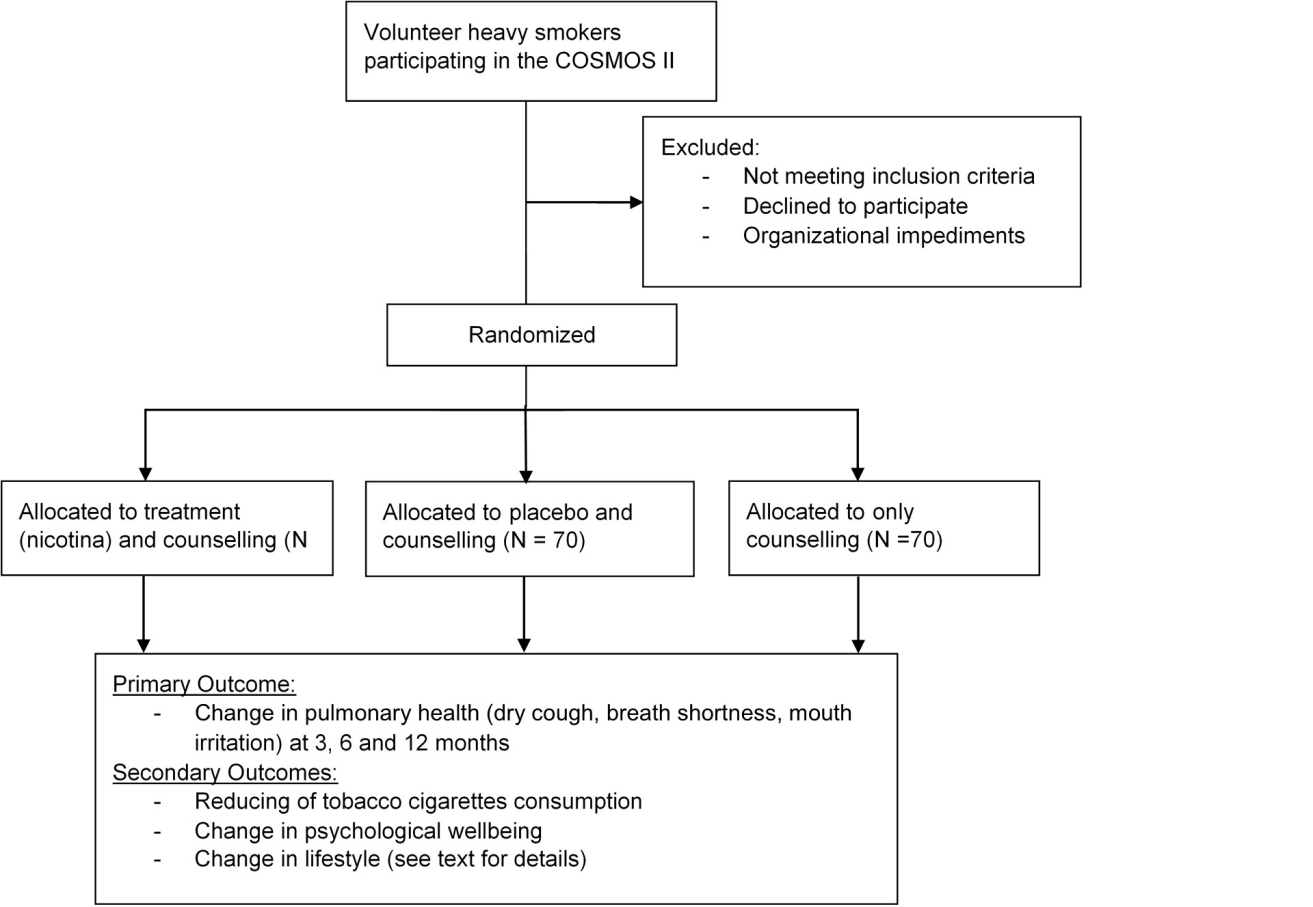
CONSORT flow diagram.

This is a prospective, randomized, placebo-controlled, double-blind, three-parallel group study. The study protocol was designed using the recommendations of the Consolidated Standards of Reporting Trials statement (Figure 1).

For this study, we opted for the VP5 electronic cigarettes kit, which offered a good quality/price ratio and proven reliability and safety. Nicotine and nicotine-free liquids are produced by BioFumo, which fully collaborated with us and provided liquids in nicotine concentrations of 8 mg/mL and packages that were not distributed for commercial use.

### Participants

Volunteer smokers are recruited from among COSMOS II participants at the IEO hospital. Details on the inclusion and exclusion criteria are provided in the “Selection Criteria” section below.

The main inclusion criterion is adult healthy smokers who voluntarily choose to take part in a lung cancer screening program. This screening program includes both a low-dose computed tomography (CT) scan and blood tests in order to detect early signs of lung cancer. Consequently, all of our participants agree to undergo these examinations and are over the age of 55 at the beginning of the study. To be included in the study, COSMOS II participants must have smoked an average of 10 cigarettes or more a day for at least the past 10 years. Furthermore, they also must report strong motivation to stop smoking as measured by a motivational questionnaire (see the “Instruments and Measures” section).

Since we are interested in assessing the effect of a specific e-cigarettes-based treatment, we exclude smokers already using e-cigarettes, which we define as smokers who had ever regularly used e-cigarettes for more than 1 week alone or in combination with tobacco cigarettes. Thus, all participants are inexperienced with the use of e-cigarettes (full instructions are provided by the researcher in charge of the study during the briefing). Also, smokers who at the moment of the interview are undergoing NRT or underwent NRT in the previous 6 months are excluded. In this way, we tried to prevent any psychological and physiological confounding effects due to previous treatments. Furthermore, people with a history of psychiatric, severe dyspnea, and cardiovascular diseases are also excluded.

All the including and excluding criteria are evaluated during the first clinical examination of the COSMOS II program. Only after the clinical examination, which includes objective tests and an anamnestic interview by a physician in charge, is a smoker considered for possible inclusion in the protocol.

### Selection Criteria

#### Inclusion Criteria

1. Subjects are involved in the COSMOS II study
2. Subjects have smoked at least ten cigarettes a day for the past 10 years
3. Subjects wish to reduce tobacco smoking (motivational score higher than 10) who are not treated at a smoking center
4. Signed informed consent

#### Exclusion Criteria

1. Symptomatic cardiovascular disease
2. Symptomatic severe respiratory disease
3. Regular psychotropic medication use
4. Current or past history of alcohol abuse
5. Use of smokeless tobacco or NRT
6. Participation in another antismoking program in the current year

### Treatments

#### Group 1 Treatment (E-Cigarette and Support)

The participants receive an e-cigarettes kit and 12 10-mL liquid cartridges containing an 8 mg/mL concentration of nicotine. They are instructed to use the electronic cigarette ad libitum during the first week before their quitting day (determined at the first contact) in order to familiarize themselves with its use. Starting at Week 2 (soon after the designated quitting day), the participants are asked to stop smoking tobacco cigarettes and use the e-cigarettes exclusively for the next 11 weeks. E-cigarette use is monitored through a weekly paper diary and regular telephone interviews. Since it is not possible to exclude tobacco-cigarette smoking after the quit day, the weekly diary also contains items related to cigarette smoking. Tobacco smoking is also addressed during the periodic calls.

During treatment, a low-intensity remote (by phone) counseling program is provided to maintain motivation and monitor any psychological and/or physical problems related to the study protocols. This program includes 4 calls in total, 1 at the ends of Week 1, Week 4, Week 8, and Week 12. Each call will last about 10 minutes; will address concerns about any ongoing psychological, physical, and behavioral changes; and will support the participants’ continued motivation by providing practical suggestions.

The participants also receive an electronic fitness bracelet in order to assess their physical activity and sleep quality during treatment.

#### Group 2 Treatment (Placebo)

The participants receive an e-cigarettes kit and 12 10-mL nicotine-free liquid cartridges. This liquid has the same manufacturer, flavor, and components (except for nicotine) as the one used in Group 1. All other procedures and measures used for Group 2 are the same as those for Group 1.

#### Group 3 Treatment (Control/Support-Only)

The participants are provided only with low-intensity remote (by phone) antismoking counseling to motivate and support cigarette smoking cessation and abstinence. Scheduling, aims, and structure are the same as those of Group 1. The participants also receive an electronic fitness bracelet in order to assess their physical activity and sleep quality during treatment. All measures and procedures are identical to Groups 1 and 2.

#### Treatments Common to All Groups

- A complete explanation of the project and informed participant consent
- Baseline behavioral, motivational, and psycho-cognitive evaluation (set of questionnaires)
- Baseline clinical parameter assessment
- Initial and final face-to-face interview
- Regular telephone interviews
- Explanation of the weekly short-diary procedure
- Briefing on and delivery of the e-bracelet
- Low-intensity remote smoking cessation counseling program

### Instruments and Measures

#### Clinical Parameter Evaluation

- Anamnesis
- Clinical examination
- CO measurement
- Low-dose CT scan
- Circulating micro-RNA examination
- Respiratory examination

#### Instruments

- Self-reported measures are used for cough and other respiratory symptoms assessment. We opted for Likert scales to measure cough, breath shortness, mouth irritation, and phlegm frequency as well as the Leicester Cough Questionnaire.
- Fagerstrom Test for Nicotine Dependence: a 6-item self-reporting questionnaire assessing nicotine dependence. It requires a few minutes to complete [12]. This test is administered to all COSMOS II participants.
- Motivational questionnaire [13]: a 4-item self-reporting questionnaire assessing motivation to quit smoking. The total classifies the patient into 1 of 4 motivational categories (from “not ready to quit” to “highly motivated”). This test is administered to all COSMOS II participants.
- Hospital Anxiety and Depression Scale (HAD): The HAD is a self-administered questionnaire composed of two 7-item scales, 1 for anxiety and 1 for depression, which should be used as 2 separate measures of emotional distress. The scale has been validated for Italian culture by Costantini and showed high internal consistency with Crohnbach alpha, ranging from .83 to .85 [14]. The HAD evaluates symptoms of anxiety and depression, avoiding misattribution due to the physical aspects of the illness. The values range from 0 to 21 for each scale. Cutoff scores are preliminarily defined as normal (0-5), mild (6-8), moderate (9-11), and severe (greater than 11) for both anxiety and depression patients [15].
- BIS/BAS Scale [16]: a 20-item self-reporting questionnaire evaluating the behavioral inhibition system (BIS) and BAS. Each of the items is rated on a 5-point scale, ranging from 1 (does not describe me at all) to 5 (describes me very well).
- Barratt Scale [17]: an 11-item self-reporting questionnaire designed to measure impulsiveness. All items are measured on a 4-point scale (Rarely/Never; Occasionally; Often; Almost Always/Always), where a 4 generally indicates the most impulsive response, although some items are scored in reverse order to avoid a response bias [18].
- The Leicester Cough Questionnaire: a valid, self-reported cough-specific health status measure. It is a 19-item questionnaire that has been validated in acute and chronic cough [19]. The overall score ranges from 3 to 21 with a higher score indicating a better quality of life.
- Electronic bracelet (the Flex FitBit): a device that allows lifestyle monitoring of physical activity and sleep characteristics, including: sleeping and napping (hours slept, light vs deep sleep, and waking periods); activity (distance, calories burned, activity time, and activity intensity); nutrition (food and beverage intake); mood (assessment of the affect state); and an insight engine that identifies hidden connections and patterns in day-to-day activities.
- The exhaled CO is measured by the The Micro+ Smokerlyzer, which has less than 5% H_2_ cross-sensitivity.
- Ad hoc questionnaire: measures demographic data, self-perceived quality of life (analogue scale), physical activity, and smoking-related issues (characteristics of the smoking experience).

### Recruitment and Follow-Up

All inclusion and exclusion criteria are checked during the registration procedure and initial assessment for inclusion in COSMOS II. Eligible participants are asked to provide informed consent. The informed consent form is signed and dated by both the participant and the physician. Enrolled participants receive a chronological number and are assigned to a treatment group (e-cigarettes with nicotine, e-cigarettes without nicotine, or control).

A randomization list using a permuted block design (40 blocks of 6 subjects randomly assigned to 1 of the 3 treatment groups) have been previously prepared by an independent personnel unit and labeled with the progressive number applied to the packaging containing e-cigarettes and liquid cartridges with or without nicotine (Group 1 and Group 2).

Neither the participant nor the researcher in charge knows whether the liquid in the e-cigarettes kit contains nicotine. Only the statistician who prepared the randomization list and the person labeling the e-cigarettes packaging know the actual treatment.

Each participant is then assigned to one of the three groups and receives the related treatment, as illustrated above.

Follow-up: 6 months (at the IEO)

- Behavioral psycho-cognitive questionnaires
- Clinical parameters assessment (respiratory symptoms)
- Smoking status assessment (questionnaire and CO level). The nonsmoking status is established by self-report items and the CO level (ppm<5).
- Debriefing, during which we also ask participants of Group 1 and Group 2 to guess if they used a nicotine-free or a nicotine-based e-cigarette.

End-point: 12 months (at the IEO, during the annual assessment of COSMOS II)

- Clinical parameter assessment (respiratory symptoms)
- Final behavioral and psycho-cognitive assessment
- Smoking status assessment (questionnaire and CO level)
- Debriefing and collection of comments

**Figure 2:**
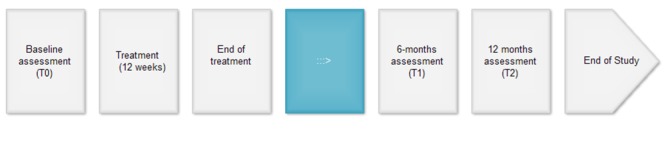
Protocol plan.

Timeline (Figure 2):

- Distribution of e-cigarettes: 3 months (ends at Week 12)
- Data tracking (e-bracelet) and active monitoring: 6 months (ends at Week 24)
- End point: 12 months

### Endpoints/Evaluation Criteria

#### Primary Outcome Measures

- Change in respiratory symptoms (eg, dry cough, breath shortness, mouth irritation)

To evaluate the impact of a 3-month e-cigarettes program to reduce smoking-related respiratory symptoms (dry cough, breath shortness, mouth irritation, and phlegm) through reduced tobacco cigarette consumption. The primary outcome is measured at Month 6 and then at the follow-up at Month 12.

#### Secondary Outcome Measures

- Change in psychological well-being (HAD scale)
- Change in number of cigarettes smoked daily
- Change in the concentration of exhaled air CO
- Change in daily activity (mean number of daily steps)
- Change in lifestyle as measured by ad-hoc questionnaires

### Statistical Considerations

#### Sample Size

Starting with data provided by previous studies on the effect of smoking discontinuation, whether using or not using e-cigarettes [2,20,21], we expected to find a reduction between 20% and 30% of respiratory symptoms reported by participants. We used cough as the measure to power the trial on. Using a two-sided *Z* test, a sample of 70 participants in either of the experimental groups (e-cigarettes with or without nicotine) and 70 in the control group (counseling alone) will reach 80% power, at .05 significance level, to detect a 20% reduction in the frequency of symptoms from the baseline in either of the e-cigarette groups (with or without nicotine) compared to a 5% reduction in the control group (counseling alone).

### Statistical Plan

The main analysis will consist of comparing the reduction in symptoms after 6 months for the three groups: e-cigarettes with nicotine vs control; e-cigarettes without nicotine vs control; and e-cigarettes with nicotine versus e-cigarettes without nicotine. Analyses will be based on two-sided *Z* tests.

A secondary analysis will assess the reduction of the consumption of tobacco-containing cigarettes after 6 months among the groups. Analysis will be based on paired Student’s t-test or the Wilcoxon signed rank tests.

No interim monitoring is planned since, given the sample size, any interim analysis would have too few events to be interpreted. Efforts will be made to maximize retention by maximizing trust at the baseline assessment and collecting multiple means to contact and commit participants, including email and phone contact details, and by assertive follow-up. Starting with our previous experience with a similar population coming from the COSMOS I program, we expect that our participants will be compliant with and committed to the aims of the study. Giving the expected intrinsic motivation of participants and thanks to the above strategies, the study aims to have at least 80% retention at 6 months and 70% at 12 months. Considering these figures, we expect to maintain a statistical power to detect a reduction of 5 cigarettes/day in our smokers (the cigarettes per day mean is about 20 in the COSMOS population). Thus, using a two-sided two-sample *t*-test with a significance level (alpha) of .05, a sample size of 49 participants per arm will achieve 80% power to detect a mean reduction of 5 cigarettes/day between any of the 2 experimental arms and the control arm, assuming a mean consumption of 20 cigarettes/day in the control arm and a common standard deviation of 8.7.

### Ethical Considerations

This study is performed in accordance with the principles stated in the Declaration of Helsinki and subsequent amendments, and in accordance with the Good Clinical Practice Guideline. This protocol was assessed and certified by the ethical board of Fondazione Umberto Veronesi, the ethical committee of Università degli studi di Milano, and the ethical committee of the European Institute of Oncology. The ClinicalTrials.gov identifier is NCT02422914. Informed consent will be obtained from all subjects.

## Results

At the time of manuscript submission, the trial’s status is “recruiting.”

## Discussion

### Principal Findings

Cigarette smoking is a major risk factor for a variety of diseases. The World Health Organization states that tobacco kills nearly 6 million people each year and that an annual death toll of more than 8 million is expected by 2030.

Despite the availability of approved medications and smoking cessation aids (ie, NRT, bupropion, varenicline, and counseling programs), long-term quitting rates are relatively low. Most smokers try to quit without professional help even when they see their doctor on a regular basis. Indeed, smoking status is rarely documented and smoking cessation treatments are offered even less frequently [22], wasting an opportunity provided by the doctor-patient relationship.

The failure of tobacco control is clear in developing countries. However, in most rich countries, tobacco control also is problematic due to a number of factors. Economic and ethical issues often conflict in antismoking research and strategies [23], and the relatively recent availability on the market of e-cigarettes has added further confusion [24]. Consequently, independent studies are needed in this field to avoid any possible external influence. Furthermore, we believe that lung cancer screening programs (like COSMOS II, where this project is nested) have an ethical obligation to provide participants access to all the information and strategies that could help them to reduce their risk. Stopping or reducing tobacco consumption, then, should be a primary goal. However, traditional antismoking programs are expensive and not always effective. Therefore, favoring a partial or complete shift to e-cigarettes in heavy smokers (eg, persons at high risk for a number of diseases) could be considered a moral imperative. However, to follow this path, sound and reliable data is required on a large sample and in a variety of contexts.

Last, the question of the use of e-cigarettes and their regulation concerns not only physiological and toxicity aspects (eg, the association between cancer and smoking), but also certain relevant behavioral and psychological aspects as well. Thus, it is necessary not only to understand the toxicity of these new ways of smoking, but also their impact on smokers’ minds and lifestyles.

### Conclusions

E-cigarettes-based intervention could provide a gateway to boosting health-related behavior changes, in order to reduce tobacco consumption and positively impact smokers’ quality of life. Indeed, reducing tobacco consumption or supporting abstinence could be considered a fundamental aim of a screening program, since a change in smoking habits reduces the risk of smoking-related diseases.

Recent studies [2,3,25] show that e-cigarettes must be considered safe devices that are potentially useful both for reducing clinical symptoms (eg, cough, phlegm, breath shortness) and enhancing the impact of antismoking interventions. Consequently, the use of e-cigarettes is particularly important in prevention programs and for high-risk subjects. Many aspects are currently unclear and debated [25]. Hence, the outcome of this study will provide important data on the possible role of e-cigarettes as tools for use in screening and prevention programs. We argue that e-cigarettes apply a medicalized substitution logic in which nicotine dependence becomes a route to health in addition to a disorder to be treated.

## Abbreviations

BAS: behavioral approach system
BIS: behavioral inhibition system
CT: computed tomography
CO: carbon monoxide
COSMOS: Continuous Observation of SMOking Subjects
HAD: Hospital Anxiety and Depression Scale
IEO: European Institute of Oncology
NRT: nicotine replacement therapy
